# Society of Homœopathicians—A Reply to Its Critics

**Published:** 1895-03

**Authors:** J. A. Biegler


### SOCIETY OF HOMŒOPATHICIANS.

### A REPLY TO ITS CRITICS.

TO THE EDITOR OF THE HOMŒOPATHIC PHYSICIAN:—In relation to the preamble to the Constitution of the Society of Homœopathicians pnblished in the last November number of THE HOMŒOPATHIC PHYSICIAN and the protest from the I. H. A., signed by Dr. Baylies as President and Dr. Allen as Vice-President, a protest signed by Dr. Fincke and an article entitled "The Society of Homœopathicians," by Dr. Butler, all published in the last January number of said journal, there is this to be said:

The part of the preamble complained of is a simple statement of facts and conclusions adduced therefrom.

The central fact—*i. e.*, that the I. H. A. voted "to indefinitely postpone a report of the Board of Censors before it had been submitted," is not controverted by either Drs. Baylies, Allen, or Butler, and is specifically admitted by Dr. Butler.

Dr. Fincke does not admit or deny anything in the preamble, but "from first to last emphatically disavows and disapproves the same," and avers that falsehoods are contained in the preamble but specifies none.

The only fact stated in the preamble attempted to be controverted by either of the parties is that the action of the I. H. A., in indefinitely postponing the report, was deliberate, and was intended to shield a member in error.

But in both the articles by Drs. Baylies and Allen and by Dr. Butler it is admitted and alleged, in the first that "the impression prevailed that the interests of the Association would best be served by omitting their consideration," and in the second "that it was the deliberate judgment of the majority that no good could come from discussion of the charges made, and its deliberate intention not only to prevent such discussion but to ignore the charges seems more than probable."

So it stands admitted by these three doctors that it was the deliberate intention of the majority of the members of the Association to squelch the report and shut off all debate on the charges to which it related, but that it was the deliberate intention to do it in the way it was done is denied. This is a mere quibble. It was deliberately intended to accomplish a certain object. The object was accomplished, and these parties now quibble over the word "deliberate" as to the *mode* of accomplishing the object deliberately intended to be accomplished.

If the squelching of the report and shutting off debate was not to shield a member in error, at least one charged with error, and who had been tried under the rules of the Association before the proper tribunal, which had made its report and was ready to submit its decision to the Association, what was the object? There could have been no other object.

The decision of the Board of Censors was ready for presentation. What the decision was was not officially known, but it was pretty generally believed to find the accused guilty on some of the charges, and for that reason it was deliberately planned to suppress it in some manner, to shield a member believed to have been adjudged in error.

Had it been generally believed that the decision did not adjudge the accused member to be in error but exonerated him from the charges, would there have been any occasion to deliberately suppress it?

The above is sufficient answer to Dr. Butler's statement that charges were unproven. They had been tried, the proofs on both sides submitted, the decision made, and all the proofs ever to be given had been given.

That the suppressing of the report of the Board of Censors and shutting off all debate of the charges in some manner was deliberately planned thus stands admitted in the articles which criticise the preamble for applying the word deliberate to such action.

That such action of the I. H. A. was a violation of its Declaration of Principles, Constitution, and By-Laws, and destroyed the life of pure Homœopathy as represented by the I. H. A. is a conclusion to be drawn from such action in conjunction with the Declaration of Principles, Constitution, and By-Laws, and is a necessary conclusion.

By the Constitution no person could become a member of the I. H. A. without indorsing its Declaration of Principles, *a priori*, no person who did not indorse and practice according to the same should remain a member.

By section 16 of the By-Laws it was provided that the Board of Censors should investigate charges in writing preferred against any member for unhomœopathic practice, or for advocating practices contrary to the Declaration of Principles, and should report to the Association the results of such investigation for final action, and that the Association, by a two-thirds vote of the members present, should have power to expel.

By indefinitely postponing the report of the Board of Censors, the result of their investigation of charges preferred against a member for unhomœopathic practice and advocating practices contrary to the Declaration of Principles, the I. H. A. violated its sixteenth By-Law by preventing the Board of Censors from performing a duty imposed on it by such By-Law; by preventing final action thereon by the Association, a duty imposed on it by such By-Law; by depriving the Association of its right to purge its body of an unworthy member by expulsion if the report of the Board of Censors should show that the member against whom the charges had been made was unworthy of membership.

The Declaration of Principles was the foundation of the Association. The Constitution was the mere organization of the Association and declarative of who were worthy of membership, and the By-Laws, rules for the regulation of meetings, modes of procedure in meetings and accepting new members, trials of delinquents and expulsion of unworthy members. A violation of the By-Law providing for final action on the report of the Board of Censors of the result of its investigation of charges against a member and the expulsion of an unworthy member, which prevented the Board of Censors presenting its report, as required, and prevented final action thereon, and prevented the Association from voting on the question of purging itself of and expelling a member, was a violation of the whole, and destroyed the life of pure Homœopathy as represented by the I. H. A., for how could any self-respecting men afterward act as a Board of Censors, spend the time and labor to investigate charges against a member with the possibility that their report should never see the light?

The most unprincipled practitioner on earth might, by deceit, become a member, but with such a precedent, and a few persistent, loud-mouthed, and unprincipled supporters, the Association could never purge itself of such a member, and would finally and surely fall entirely under the control of the unprincipled pretenders, wolves clothed with wool and the name of Hahnemannians.

J. A. Biegler.

